# Hypoxic hepatitis after cardiac arrest is associated with 30-day mortality, poor neurological outcome and non-occlusive mesenteric ischemia

**DOI:** 10.1016/j.resplu.2026.101472

**Published:** 2026-08-31

**Authors:** Peter Hunyady, Asieb Sekandarzad, Dawid L. Staudacher, Tobias Wengenmayer, Hendrik Luxenburger, Robert Thimme, Katharina Mueller-Peltzer, Alexander Supady, Dominik Bettinger

**Affiliations:** a Interdisciplinary Medical Intensive Care, Medical Center – University of Freiburg, Faculty of Medicine, University of Freiburg, Freiburg, Germany; b Department of Medicine II: Gastroenterology, Hepatology, Endocrinology and Infectiology, Medical Center – University of Freiburg, Faculty of Medicine, University of Freiburg, Freiburg, Germany; c Department of Radiology, Medical Center – University of Freiburg, Faculty of Medicine, University of Freiburg, Freiburg, Germany

**Keywords:** Cardiac arrest, Post-resuscitation care, Extracorporeal cardiopulmonary resuscitation, Hypoxic hepatitis, Non-occlusive mesenteric ischemia

## Abstract

**Background and aims:**

Abdominal organ dysfunction after cardiac arrest may reflect hepatosplanchnic hypoperfusion. Hypoxic hepatitis may indicate severe systemic hypoperfusion and a hepatosplanchnic ischemic phenotype. We assessed hypoxic hepatitis occurrence and associations with 30-day mortality, favourable neurological outcome and non-occlusive mesenteric ischemia after conventional and extracorporeal cardiopulmonary resuscitation (CPR, ECPR).

**Methods:**

We performed a retrospective single-centre cohort study of adult patients admitted after cardiac arrest between 11/2020 and 02/2026. Hypoxic hepatitis was defined as peak aspartate or alanine aminotransferase ≥20 times the sex-specific upper limit of normal within 72 h after collapse, after exclusion of primary non-hypoxic hepatopathy. Analyses were restricted to patients alive at 72 h and performed separately for CPR and ECPR using adjusted Cox proportional hazards and logistic regression models.

**Results:**

Among 835 patients, hypoxic hepatitis occurred in 58/702 CPR patients (8.3%) and 29/133 ECPR patients (21.8%). In the CPR cohort, hypoxic hepatitis was associated with higher 30-day mortality (adjusted HR 1.63, 95% CI 1.11–2.42), lower odds of favourable neurological survival (adjusted OR 0.33, 95% CI 0.17–0.64) and higher odds of non-occlusive mesenteric ischemia (adjusted OR 7.02, 95% CI 2.61–18.89). Corresponding adjusted estimates in ECPR patients were HR 2.65 (95% CI 1.44–4.90), OR 0.09 (95% CI 0.02–0.33) and OR 3.99 (95% CI 1.35–11.78), respectively.

**Conclusion:**

Among patients alive 72 h after cardiac arrest, hypoxic hepatitis was associated with increased 30-day mortality, poor neurological outcome and non-occlusive mesenteric ischemia and may help identify a hepatosplanchnic high-risk phenotype after both CPR and ECPR.

## Introduction

Cardiac arrest remains a major cause of death and disability despite advances in resuscitation and post-resuscitation care.^1^ After return of spontaneous circulation (ROSC), post-cardiac arrest syndrome contributes to mortality through brain injury, myocardial dysfunction, systemic inflammation, vasoplegia and multi-organ failure.^2^, ^3^, ^4^ Markers of global ischemia and organ hypoperfusion may therefore add prognostic information beyond arrest-related characteristics.^2^, ^4^, ^5^, ^6^, ^7^ Hepatic oxygen delivery depends on arterial and portal venous flow, both of which are reduced during low-flow states and splanchnic vasoconstriction.^8^, ^9^ Hypoxic hepatitis, also termed ischemic hepatitis or “shock liver”, is defined by a rapid and marked increase in serum aminotransferases caused by impaired oxygen delivery and reduced hepatic blood flow.^8^, ^10^

In critically ill patients, hypoxic hepatitis usually accompanies circulatory shock or severe respiratory failure and may reflect systemic ischemia–reperfusion injury after cardiac arrest rather than isolated liver disease.^9^, ^11^, ^12^, ^13^, ^14^, ^15^ It has been associated with longer no-flow or low-flow durations, higher lactate levels, post-resuscitation circulatory shock, worse neurological recovery and increased mortality.^13^, ^14^, ^15^, ^16^, ^17^ Conventional cardiopulmonary resuscitation (CPR) and extracorporeal cardiopulmonary resuscitation (ECPR) represent distinct post-arrest populations. ECPR restores systemic perfusion in selected patients with refractory cardiac arrest but is typically preceded by prolonged low-flow duration and involves altered regional perfusion and oxygenation, anticoagulation and other features of mechanical circulatory support.^18^, ^19^, ^20^ These differences may influence the occurrence and prognostic relevance of hypoxic hepatitis, but comparative CPR and ECPR data remain scarce.^14^, ^18^, ^19^, ^20^ The splanchnic circulation is likewise vulnerable to low-flow states and vasoconstriction after cardiac arrest.^21^, ^22^ Non-occlusive mesenteric ischemia, caused by mesenteric hypoperfusion and vasospasm without major arterial or venous occlusion, is a life-threatening post-arrest complication that may be difficult to detect early in sedated patients because clinical signs are non-specific.^21^, ^22^, ^23^, ^24^, ^25^, ^26^, ^27^, ^28^ Because the liver and intestines are highly perfusion-dependent, hypoxic hepatitis may identify a hepatosplanchnic ischemic phenotype involving global hypoperfusion, impaired regional reperfusion and non-occlusive mesenteric ischemia.^23^, ^24^, ^25^, ^26^ However, this association remains poorly defined across conventional CPR and ECPR cohorts.^23^, ^24^, ^26^

To address this gap, this study aimed to examine the occurrence of hypoxic hepatitis and its associations with 30-day mortality, favourable neurological outcome and non-occlusive mesenteric ischemia among patients surviving the first 72 h after either conventional CPR or ECPR.

## Patients and methods

### Study design and participants

**Fig. 1:**
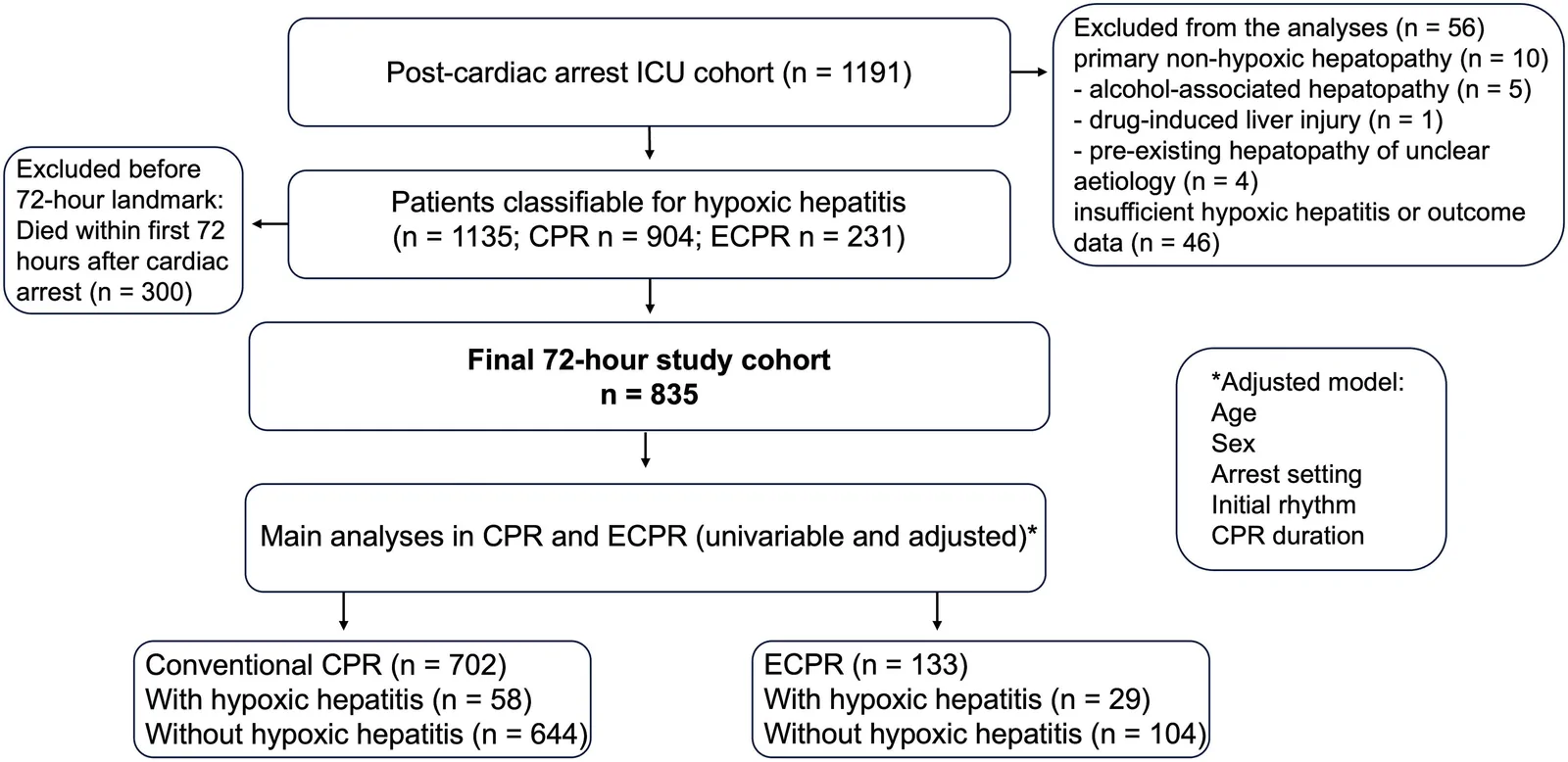
**Flowchart of patient selection and final study cohort**.

We performed a retrospective single-centre cohort study of all consecutive adult patients admitted to the Interdisciplinary Medical Intensive Care Unit of the University Medical Center Freiburg (Germany) after cardiac arrest between November 2020 and February 2026. Patients were identified by medical coding and electronic-record review. Eligible patients achieved ROSC after conventional CPR or underwent ECPR, defined as initiation of veno-arterial extracorporeal membrane oxygenation during ongoing chest compressions for refractory cardiac arrest. We excluded patients with insufficient data for hypoxic hepatitis classification or outcome assessment and those with evidence of primary non-hypoxic hepatopathy as the main cause of liver injury. In accordance with the 72-h landmark design, patients who died within the first 72 h after cardiac arrest were excluded from the final study cohort. The study flow is shown in Fig. 1. This study is reported in accordance with the Strengthening the Reporting of Observational Studies in Epidemiology (STROBE) statement.^29^

### Definitions and data collection

Hypoxic hepatitis was defined as a peak aspartate aminotransferase (AST) or alanine aminotransferase (ALT) level of at least 20 times the sex-specific upper limit of normal (ULN) within 72 h after collapse. Primary non-hypoxic hepatopathy was excluded by a consulting hepatologist blinded to study outcomes using pre-existing clinical and laboratory information.^8^, ^9^ For both AST and ALT, the ULN was 50 U/L in men and 35 U/L in women. Non-occlusive mesenteric ischemia was defined as mesenteric ischemia diagnosed during hospitalisation without occlusion of the main arterial splanchnic branches, confirmed by computed tomography, endoscopy or surgical findings. The final diagnosis of non-occlusive mesenteric ischemia was confirmed by an experienced ICU physician blinded to mortality and neurological outcomes.^21^, ^22^, ^23^, ^24^, ^25^, ^26^, ^27^

Initial rhythm was classified as shockable or non-shockable and arrest setting as out-of-hospital (OHCA) or in-hospital cardiac arrest (IHCA). CPR duration was defined as the time from initiation of resuscitation by bystanders or professional rescuers, whichever occurred first, until sustained ROSC or initiation of extracorporeal circulation. No-flow time was the estimated interval from collapse to initiation of resuscitation and was recorded as missing for unwitnessed arrests without a reliable estimate. Initial lactate was defined as the first arterial blood lactate concentration measured after collapse. At our centre, ECPR is considered for refractory cardiac arrest when ongoing resuscitation is judged effective and extracorporeal circulation is expected within 60 min. Witnessed collapse or a reliably presumed short no-flow interval is considered favourable but is not applied as an absolute criterion. Advanced age and relevant comorbidities are considered very cautiously in the decision for ECPR.^28^

### Study outcomes

The primary outcome was 30-day mortality. Patients discharged alive without clinical deterioration before day 30 were considered alive at 30 days. Secondary outcomes were 30-day survival with favourable neurological outcome, defined as Cerebral Performance Category (CPC) 1–2, and detection of non-occlusive mesenteric ischemia during the index hospitalisation.^30^ Mortality was analysed as a time-to-event endpoint from 72 h after cardiac arrest to day 30, whereas favourable neurological outcome and non-occlusive mesenteric ischemia were analysed as binary outcomes.

Clinical, resuscitation-related, laboratory and outcome data were retrospectively extracted from electronic medical records, including demographics, arrest characteristics, resuscitation time intervals, pre-existing liver cirrhosis, arterial blood gas and liver laboratory parameters, non-occlusive mesenteric ischemia diagnosis and management, 30-day mortality and neurological outcome.

### Statistical analysis

Baseline characteristics are described separately for CPR and ECPR according to hypoxic hepatitis status. Continuous variables are reported as median with interquartile range and categorical variables as counts and percentages. Groups were compared using Mann–Whitney U tests for continuous variables and chi-square or Fisher’s exact tests for categorical variables. Associations of hypoxic hepatitis with 30-day mortality were assessed using Kaplan–Meier analysis, log-rank testing and Cox proportional hazards regression; associations with favourable neurological survival and non-occlusive mesenteric ischemia were assessed using logistic regression. Each endpoint was examined in univariable analysis and one prespecified multivariable model adjusted for age, sex, initial rhythm, arrest setting and CPR duration.

To evaluate whether hypoxic hepatitis provided prognostic information beyond established markers of ischemic burden, exploratory models for 30-day mortality, favourable neurological survival and non-occlusive mesenteric ischemia additionally included either no-flow time or initial lactate. Hepatic injury severity was also modelled continuously as the log2-transformed maximum of AST/ULN or ALT/ULN, with effect estimates representing the change per doubling of transaminase burden. For visualisation, adjusted predicted probabilities for 30-day mortality, favourable neurological outcome and non-occlusive mesenteric ischemia across the observed range of log2-transformed transaminase burden were derived from the respective multivariable models separately for CPR and ECPR patients.

Missing data were not imputed and all multivariable analyses were complete-case analyses. Tests were two-sided with *p* < 0.05 considered statistically significant. Statistical analyses were performed using IBM SPSS Statistics version 31.0.2.0.

### Ethical approval

The study was conducted in accordance with the Declaration of Helsinki and applicable institutional and national regulations and was approved by the Ethics Committee of the University of Freiburg on 20 May 2026 (approval number 26-1162-S1-retro).

## Results

### Study cohort and baseline characteristics

Of 1191 patients admitted after cardiac arrest, 56 were excluded: 46 had insufficient data for classification or outcome assessment and 10 had primary non-hypoxic hepatopathy (five alcohol-associated, one drug-induced and four pre-existing hepatopathies of unclear aetiology without a definitive pre-arrest diagnosis). Among the remaining 1135 patients, 300 patients (26.4%) died within the first 72 h after cardiac arrest and were not included in the study cohort.

The final study cohort comprised 835 patients. Of these, 702 patients (84.1%) had been treated with conventional CPR and 133 patients (15.9%) with ECPR. Hypoxic hepatitis was present in 87/835 patients (10.4%), 58/702 patients (8.3%) in the CPR subgroup and 29/133 patients (21.8%) in the ECPR subgroup. Baseline characteristics are summarised in Table 1. No-flow time was unavailable in 64/835 patients (7.7%), including 58/702 conventional CPR patients (8.3%) and 6/133 ECPR patients (4.5%).

**Table 1:** Baseline characteristics according to hypoxic hepatitis status.

	**CPR**				**ECPR**			
	**Overall** **(*n* = 702)**	**Without hypoxic hepatitis** **(*n* = 644)**	**Hypoxic hepatitis** **(*n* = 58)**	***P* value**	**Overall** **(*n* = 133)**	**Without hypoxic hepatitis** **(*n* = 104)**	**Hypoxic hepatitis** **(*n* = 29)**	***P* value**
Age, years	68.1 (59.5–76.5)	68.5 (59.5–76.9)	66.5 (60.4–72.7)	0.151	60.6 (51.7–68.0)	61.4 (52.7–68.5)	57.6 (43.2–68.0)	0.235
Male sex, *n*/*N* (%)	497/702 (70.8%)	458/644 (71.1%)	39/58 (67.2%)	0.534	95/133 (71.4%)	79/104 (76.0%)	16/29 (55.2%)	0.028
In-hospital cardiac arrest, *n*/*N* (%)	255/702 (36.3%)	226/644 (35.1%)	29/58 (50.0%)	0.024	80/133 (60.2%)	63/104 (60.6%)	17/29 (58.6%)	0.849
Witnessed arrest, *n*/*N* (%)	598/702 (85.2%)	554/644 (86.0%)	44/58 (75.9%)	0.037	124/133 (93.2%)	96/104 (92.3%)	28/29 (96.6%)	0.683
Shockable initial rhythm, *n*/*N* (%)	350/687 (50.9%)	330/629 (52.5%)	20/58 (34.5%)	0.009	56/132 (42.4%)	48/103 (46.6%)	8/29 (27.6%)	0.067
No-flow time, min	0.0 (0.0–2.0)	0.0 (0.0–2.0)	0.0 (0.0–2.0)	0.512	0.0 (0.0–0.0)	0.0 (0.0–0.0)	0.0 (0.0–0.0)	0.488
CPR duration, min	10.0 (5.0–20.0)	10.0 (4.0–19.0)	15.0 (8.0–29.0)	<0.001	41.0 (27.0–65.0)	40.0 (25.0–65.0)	47.0 (35.5–70.0)	0.059
Pre-existing liver cirrhosis, *n*/*N* (%)	29/702 (4.1%)	23/644 (3.6%)	6/58 (10.3%)	0.026	2/133 (1.5%)	2/104 (1.9%)	0/29 (0.0%)	>0.999
Initial lactate, mmol/L	4.4 (2.1–6.9)	4.1 (2.0–6.5)	8.6 (4.8–12.6)	<0.001	9.6 (5.8–12.4)	9.0 (5.2–11.4)	13.8 (9.7–17.0)	<0.001

Values are presented as median (interquartile range) for continuous variables and *n*/*N* (%) for categorical variables. *P* values were calculated using Mann–Whitney U tests for continuous variables and chi-square or Fisher’s exact tests for categorical variables, as appropriate.

In the CPR subgroup, patients with hypoxic hepatitis were more likely to have had an in-hospital cardiac arrest (29/58, 50.0% vs. 226/644, 35.1%; *p* = 0.024), less often had a witnessed arrest (44/58, 75.9% vs. 554/644, 86.0%; *p* = 0.037) or a shockable initial rhythm (20/58, 34.5% vs. 330/629, 52.5%; *p* = 0.009). CPR duration was longer in patients with hypoxic hepatitis (15.0 [8.0–29.0] vs. 10.0 [4.0–19.0] min; *p* < 0.001), initial lactate levels were higher (8.6 [4.8–12.6] vs. 4.1 [2.0–6.5] mmol/L; *p* < 0.001) and pre-existing liver cirrhosis was more frequent (6/58, 10.3% vs. 23/644, 3.6%; *p* = 0.026). Age, sex and no-flow time did not differ significantly between patients with and without hypoxic hepatitis.

In the ECPR subgroup, patients with hypoxic hepatitis were more often female (13/29, 44.8% vs. 25/104, 24.0%; *p* = 0.028) and had higher initial lactate levels (13.8 [9.7–17.0] vs. 9.0 [5.2–11.4] mmol/L; *p* < 0.001). Age, shockable initial rhythm, CPR duration, arrest setting, witnessed arrest, no-flow time and pre-existing liver cirrhosis did not differ significantly between patients with and without hypoxic hepatitis.

### Association of hypoxic hepatitis with mortality, neurological outcome and non-occlusive mesenteric ischemia

**Fig. 2:**
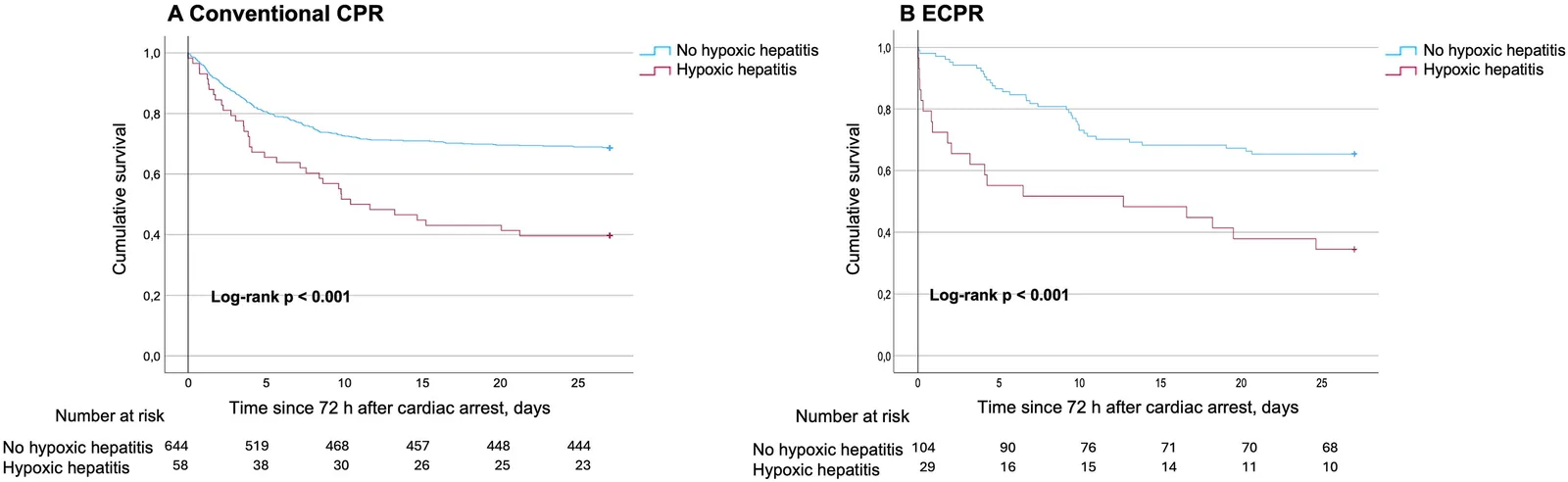
**Kaplan–Meier curves for survival according to hypoxic hepatitis status in CPR and ECPR patients. (A) Conventional CPR and (B) ECPR. Time zero was set at 72 h after cardiac arrest. Survival distributions were compared using the log-rank test; both *p* values were <0.001. Numbers at risk are displayed below each panel**.

In the CPR subgroup, hypoxic hepatitis within the first 72 h was associated with higher subsequent 30-day mortality. Mortality occurred in 35/58 patients with hypoxic hepatitis (60.3%) compared with 202/644 patients without hypoxic hepatitis (31.4%; *p* < 0.001). Kaplan–Meier estimated survival through day 30 was significantly lower in patients with hypoxic hepatitis than in those without hypoxic hepatitis (log-rank *p* < 0.001; Fig. 2A). In Cox regression, hypoxic hepatitis was associated with mortality in the univariable model (HR 2.26, 95% CI 1.58–3.23; *p* < 0.001) and after adjustment for age, sex, initial rhythm, arrest setting and CPR duration (adjusted HR 1.63, 95% CI 1.11–2.42; *p* = 0.014).

Favourable neurological outcome occurred in 17/58 patients with hypoxic hepatitis (29.3%) compared with 410/644 patients without hypoxic hepatitis (63.7%; *p* < 0.001). The association remained significant in both univariable analysis (OR 0.24, 95% CI 0.13–0.43; *p* < 0.001) and after adjustment (adjusted OR 0.33, 95% CI 0.17–0.64; *p* < 0.001). Non-occlusive mesenteric ischemia occurred in 10/58 patients with hypoxic hepatitis (17.2%) compared with 17/644 patients without hypoxic hepatitis (2.6%; *p* < 0.001). Hypoxic hepatitis was associated with increased odds of non-occlusive mesenteric ischemia in the univariable model (OR 7.68, 95% CI 3.34–17.70; *p* < 0.001) and after adjustment (adjusted OR 7.02, 95% CI 2.61–18.89; *p* < 0.001).

In the ECPR subgroup, hypoxic hepatitis was likewise associated with increased subsequent 30-day mortality. Mortality occurred in 19/29 patients with hypoxic hepatitis (65.5%) compared with 36/104 patients without hypoxic hepatitis (34.6%; *p* = 0.003). Kaplan–Meier estimated survival through day 30 was significantly lower in patients with hypoxic hepatitis than in those without hypoxic hepatitis (log-rank *p* < 0.001; Fig. 2B). Hypoxic hepatitis was associated with mortality in the univariable model (HR 2.69, 95% CI 1.54–4.70; *p* < 0.001) and after adjustment (adjusted HR 2.65, 95% CI 1.44–4.90; *p* = 0.002).

In the ECPR subgroup, favourable neurological outcome occurred in 3/29 patients with hypoxic hepatitis (10.3%) compared with 58/104 patients without hypoxic hepatitis (55.8%; *p* < 0.001). Hypoxic hepatitis was associated with markedly lower odds of favourable neurological survival in the univariable model (OR 0.09, 95% CI 0.03–0.32; *p* < 0.001) and after adjustment (adjusted OR 0.09, 95% CI 0.02–0.33; *p* < 0.001). Non-occlusive mesenteric ischemia occurred in 10/29 patients with hypoxic hepatitis (34.5%) compared with 16/104 patients without hypoxic hepatitis (15.4%; *p* = 0.022). Hypoxic hepatitis was associated with increased odds of non-occlusive mesenteric ischemia in the univariable model (OR 2.89, 95% CI 1.14–7.36; *p* = 0.026) and after adjustment (adjusted OR 3.99, 95% CI 1.35–11.78; *p* = 0.012). Results of the adjusted analyses are summarised in Table 2.

**Table 2:** Association of hypoxic hepatitis with 30-day mortality, favourable neurological outcome and non-occlusive mesenteric ischemia.

	**CPR**				**ECPR**			
	**Without hypoxic hepatitis** **(*n* = 644)**	**Hypoxic hepatitis** **(*n* = 58)**	**Unadjusted**	**Adjusted**	**Without hypoxic hepatitis** **(*n* = 104)**	**Hypoxic hepatitis** **(*n* = 29)**	**Unadjusted**	**Adjusted**
30-day mortality	202/644 (31.4%)	35/58 (60.3%)	HR 2.26 (1.58–3.23) *p* < 0.001	aHR 1.63 (1.11–2.42) *p* = 0.014	36/104 (34.6%)	19/29 (65.5%)	HR 2.69 (1.54–4.70) *p* < 0.001	aHR 2.65 (1.44–4.90) *p* = 0.002
Favourable neurological outcome	410/644 (63.7%)	17/58 (29.3%)	OR 0.24 (0.13–0.43) *p* < 0.001	aOR 0.33 (0.17–0.64) *p* < 0.001	58/104 (55.8%)	3/29 (10.3%)	OR 0.09 (0.03–0.32) *p* < 0.001	aOR 0.09 (0.02–0.33) *p* < 0.001
Non-occlusive mesenteric ischemia	17/644 (2.6%)	10/58 (17.2%)	OR 7.68 (3.34–17.70) *p* < 0.001	aOR 7.02 (2.61–18.89) *p* < 0.001	16/104 (15.4%)	10/29 (34.5%)	OR 2.89 (1.14–7.36) *p* = 0.026	aOR 3.99 (1.35–11.78) *p* = 0.012

Values are presented as *n*/*N* (%). Hazard ratios were calculated using Cox proportional hazards regression for 30-day mortality. Odds ratios were calculated using logistic regression for favourable neurological outcome and non-occlusive mesenteric ischemia. Adjusted models included age, sex, initial rhythm, arrest setting and CPR duration.

### Exploratory analyses

In exploratory models additionally including no-flow time, the association between hypoxic hepatitis and adverse outcomes remained broadly consistent. Hypoxic hepatitis remained associated with mortality in both CPR patients (adjusted HR 1.57, 95% CI 1.01–2.45; *p* = 0.048) and ECPR patients (adjusted HR 3.04, 95% CI 1.62–5.71; *p* < 0.001). Similar findings were observed for favourable neurological outcome in CPR patients (adjusted OR 0.33, 95% CI 0.17–0.66; *p* = 0.002) and ECPR patients (adjusted OR 0.08, 95% CI 0.02–0.31; *p* < 0.001), as well as for non-occlusive mesenteric ischemia in CPR patients (adjusted OR 4.26, 95% CI 1.32–13.76; *p* = 0.015) and ECPR patients (adjusted OR 5.70, 95% CI 1.74–18.72; *p* = 0.004).

In exploratory models additionally including initial lactate, the association between hypoxic hepatitis and outcomes was partly attenuated. In the CPR subgroup, hypoxic hepatitis remained associated with lower odds of favourable neurological survival (adjusted OR 0.48, 95% CI 0.24–0.95; *p* = 0.034) and higher odds of non-occlusive mesenteric ischemia (adjusted OR 4.81, 95% CI 1.70–13.64; *p* = 0.003), but the association with mortality was no longer statistically significant (adjusted HR 1.15, 95% CI 0.76–1.73; *p* = 0.508). In the ECPR subgroup, hypoxic hepatitis remained associated with mortality (adjusted HR 2.27, 95% CI 1.19–4.34; *p* = 0.013) and lower odds of favourable neurological survival (adjusted OR 0.11, 95% CI 0.03–0.42; *p* = 0.001), whereas the association with non-occlusive mesenteric ischemia was attenuated after additional adjustment for initial lactate (adjusted OR 2.01, 95% CI 0.58–6.92; *p* = 0.268).

**Fig. 3:**
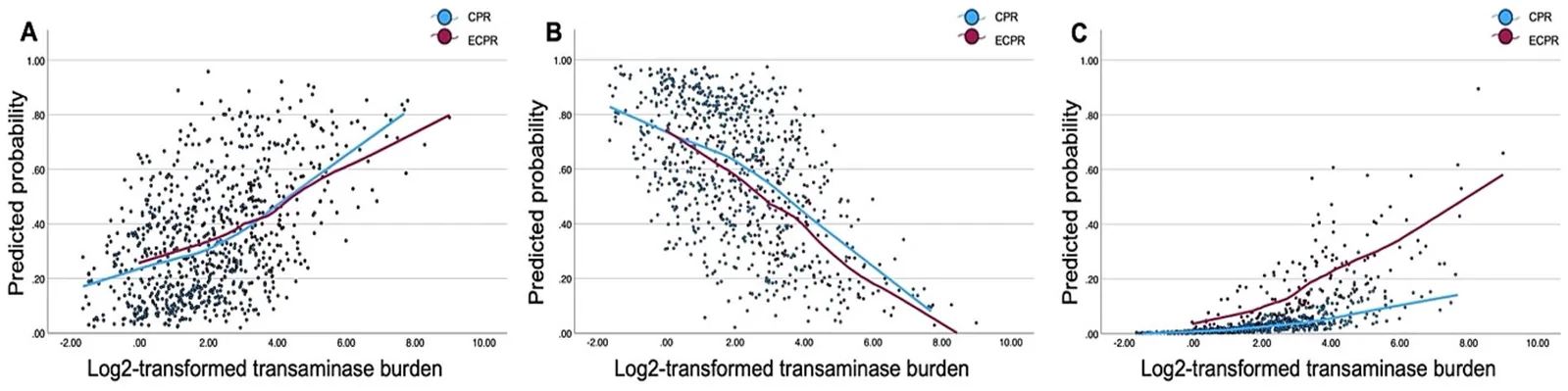
**Exploratory analysis of adjusted predicted probabilities of 30-day mortality (A), 30-day survival with favourable neurological outcome (B), and non-occlusive mesenteric ischemia (C) according to log2-transformed transaminase burden. Predicted probabilities are shown separately for CPR and ECPR patients and were derived from multivariable models adjusted for age, sex, initial rhythm, arrest setting and CPR duration**.

When hepatic injury was modelled continuously as log2-transformed transaminase burden, greater transaminase elevation was associated with adverse outcomes in both groups. In Cox regression, each doubling of transaminase burden was associated with higher mortality in CPR patients (adjusted HR 1.21, 95% CI 1.12–1.30; *p* < 0.001) and ECPR patients (adjusted HR 1.32, 95% CI 1.12–1.56; *p* = 0.001). In logistic regression, each doubling was associated with lower odds of favourable neurological survival in CPR patients (adjusted OR 0.72, 95% CI 0.64–0.81; *p* < 0.001) and ECPR patients (adjusted OR 0.58, 95% CI 0.43–0.77; *p* < 0.001), and with higher odds of non-occlusive mesenteric ischemia in CPR patients (adjusted OR 1.70, 95% CI 1.32–2.20; *p* < 0.001) and ECPR patients (adjusted OR 1.62, 95% CI 1.18–2.22; *p* = 0.003). Adjusted predicted probabilities across the observed range of transaminase burden are shown in Fig. 3.

## Discussion

In this single-centre cohort of patients who survived the first 72 h after cardiac arrest, hypoxic hepatitis was observed after conventional CPR and ECPR. Hypoxic hepatitis was associated with higher 30-day mortality, lower likelihood of favourable neurological survival and higher odds of non-occlusive mesenteric ischemia. These associations persisted after multivariable adjustment, while greater transaminase increase showed a graded association with adverse outcomes in exploratory continuous analyses.

These findings are consistent with the concept of post-cardiac arrest syndrome as a systemic ischemia–reperfusion disorder with secondary multi-organ dysfunction.^2^, ^3^, ^4^, ^5^, ^6^, ^7^ Marked aminotransferase elevation likely reflects global hypoxia, circulatory failure and reperfusion injury rather than an isolated hepatic disorder, supporting hypoxic hepatitis as an integrated marker of illness severity after resuscitation.^8^, ^9^, ^11^, ^12^, ^14^, ^15^, ^16^, ^17^, ^31^ Importantly, hypoxic hepatitis should be interpreted as an early post-arrest organ injury marker rather than as a causal baseline exposure.

The frequency of hypoxic hepatitis in our cohort was broadly consistent with previous reports in resuscitated cardiac arrest populations.^13^, ^14^, ^15^, ^16^, ^17^ Our findings extend this literature by assessing hypoxic hepatitis separately after conventional CPR and ECPR. Hypoxic hepatitis was more frequent in the ECPR cohort than in the CPR cohort. However, CPR and ECPR patients constitute clinically distinct post-arrest cohorts characterised by divergent arrest characteristics, treatment trajectories and haemodynamic profiles. Accordingly, these cohorts were analysed separately and no causal inference regarding CPR versus ECPR as a determinant of hypoxic hepatitis can be drawn from this comparison.^18^, ^19^, ^20^

A particularly important finding is the association between hypoxic hepatitis and non-occlusive mesenteric ischemia after CPR and ECPR. Non-occlusive mesenteric ischemia is a severe yet often overlooked and underdiagnosed complication after cardiac arrest and extracorporeal support.^21^, ^22^, ^23^, ^24^, ^25^, ^26^, ^27^, ^28^, ^32^, ^33^ The liver and the intestines share vulnerability to hypoperfusion, vasoconstriction and impaired reperfusion. Therefore, hypoxic hepatitis may represent a clinically accessible marker of a hepatosplanchnic ischemia–reperfusion phenotype associated with mesenteric ischemic complications.^8^, ^9^, ^10^, ^11^, ^12^, ^21^, ^22^, ^27^, ^34^, ^35^

The 72-h landmark design was chosen to address the temporal sequence between hypoxic hepatitis ascertainment and subsequent outcome assessment. Because hypoxic hepatitis develops during the early post-arrest phase, analyses starting at cardiac arrest may be affected by differential early mortality and incomplete hypoxic hepatitis ascertainment. Accordingly, mortality was analysed only after hypoxic hepatitis status had been determined.

Clinically, hypoxic hepatitis may therefore support early bedside risk stratification after cardiac arrest. Assessment of aminotransferases is inexpensive and routinely available, including for repeated measurements in intensive care practice.^10^, ^13^, ^14^, ^15^, ^16^, ^17^, ^31^, ^36^ Although hypoxic hepatitis itself is currently not a therapeutic target and should not be used in isolation to guide treatment limitation or withdrawal of care, its occurrence may justify closer haemodynamic surveillance, optimization of systemic and regional perfusion and greater diagnostic attention to abdominal complications such as non-occlusive mesenteric ischemia.^21^, ^22^, ^23^, ^24^, ^25^, ^26^, ^27^

This study has several strengths, including separate evaluation of CPR and ECPR cohorts, assessment of mortality and neurological outcome as established outcome domains in resuscitation research, inclusion of non-occlusive mesenteric ischemia as a clinically meaningful secondary endpoint, use of a 72-h study cohort and continuous transaminase modelling.^30^ Limitations include the retrospective single-centre design, residual confounding, complete-case analysis and possible detection bias for non-occlusive mesenteric ischemia due to differences in clinical suspicion and access to imaging, endoscopy or surgery.^21^, ^22^, ^23^, ^24^, ^25^, ^26^, ^27^ Furthermore, exploratory adjustment for initial lactate attenuated some associations, suggesting overlap between hypoxic hepatitis and established markers of ischemic burden. Finally, although the landmark design reduces bias from early mortality and incomplete hypoxic hepatitis ascertainment, it restricts the analysis to 72-h survivors and may introduce survivor selection.

## Conclusion

Hypoxic hepatitis after cardiac arrest was associated with increased mortality, poor neurological outcome and non-occlusive mesenteric ischemia among patients who survived to the 72-h landmark. These findings support hypoxic hepatitis as a marker of severe post-arrest hypoperfusion and possible hepatosplanchnic ischemia–reperfusion injury after both conventional CPR and ECPR. Prospective studies should validate these findings and assess their clinical implications.

## Authors’ consent

All authors have approved the final version of the manuscript and agree to be accountable for all aspects of the work.

## Data availability

The data underlying this study are not publicly available because they contain patient-level clinical information and are subject to institutional and data protection regulations. Aggregated data may be made available from the corresponding author upon reasonable request and approval by the responsible institutional authorities.

## CRediT authorship contribution statement

**Peter Hunyady:** Writing – original draft, Visualization, Methodology, Investigation, Formal analysis, Data curation, Conceptualization. **Asieb Sekandarzad:** Writing – review & editing, Conceptualization. **Dawid L. Staudacher:** Writing – review & editing. **Tobias Wengenmayer:** Writing – review & editing. **Hendrik Luxenburger:** Writing – review & editing. **Robert Thimme:** Writing – review & editing. **Katharina Mueller-Peltzer:** Writing – review & editing. **Alexander Supady:** Writing – review & editing, Supervision, Methodology, Conceptualization. **Dominik Bettinger:** Writing – review & editing, Supervision, Methodology, Conceptualization.

## Funding

We acknowledge support by the Open Access Publication Fund of the University of Freiburg.

## Declaration of competing interest

Peter Hunyady, Asieb Sekandarzad, Dawid L. Staudacher, Tobias Wengenmayer, Hendrik Luxenburger, Robert Thimme and Katharina Mueller-Peltzer declare no conflicts of interest.

Dominik Bettinger reports lecture fees from Falk Foundation and W. L. Gore & Associates, travel grants from AbbVie and research grants from the Dr. Rolf M. Schwiete Foundation and the German Research Foundation (DFG), all outside the submitted work.

Alexander Supady reports research grants and lecture honoraria from CytoSorbents Europe and Getinge, lecture honoraria from AstraZeneca, J&J and Resuscitec, travel support and consulting fees from ARTCLINE and consulting fees from LAUDA Medical and Getinge, all outside the submitted work. He is the principal investigator of the PRIME study, funded by a research grant from the German Research Foundation (DFG, project number 554212787).

The authors declare that they have no conflicts of interest directly related to the submitted work.

## Appendix A Supplementary material

Supplementary Data 1 1. STROBE checklist 2. Full models.
